# One Health Aotearoa: a transdisciplinary initiative to improve human, animal and environmental health in New Zealand

**DOI:** 10.1186/s42522-020-0011-0

**Published:** 2020-01-31

**Authors:** Sarah Harrison, Michael G. Baker, Jackie Benschop, Russell G. Death, Nigel P. French, Garth Harmsworth, Robin J. Lake, Iain L. Lamont, Patricia C. Priest, James E. Ussher, David R. Murdoch

**Affiliations:** 1 Department of Preventive and Social Medicine, University of Otago, Dunedin, New Zealand; 2 Department of Public Health, University of Otago, Wellington, New Zealand; 3 Molecular Epidemiology and Public Health Laboratory, Massey University, Palmerston North, New Zealand; 4 School of Agriculture and the Environment, Massey University, Palmerston North, New Zealand; 5 Manaaki Whenua Landcare Research, Palmerston North, New Zealand; 6 Institute of Environmental Science and Research, Christchurch, New Zealand; 7 Department of Biochemistry, University of Otago, Dunedin, New Zealand; 8 Department of Microbiology and Immunology, University of Otago, Dunedin, New Zealand; 9 Department of Pathology and Biomedical Science, University of Otago, P.O. Box 4345, Christchurch, 8140 New Zealand

**Keywords:** One health, New Zealand, Ecosystem, Indigenous, Infectious diseases

## Abstract

There is increased recognition that complex health challenges at the human-animal-environmental interface require a transdisciplinary, “*whole-of-society*” approach. This philosophy is particularly pertinent in Aotearoa-New Zealand because of the country’s relatively isolated island ecosystem, economic reliance on agriculture and its intensification, and existing indigenous worldview that emphasises holism and interconnectivity between humans, animals and the environment. In New Zealand, the One Health Aotearoa (OHA) alliance was established in order to better connect researchers and to address a growing number of infectious diseases challenges. The emphasis of OHA is to bring together and facilitate interactions between people from diverse disciplines, link to stakeholders and communities, and engage with policy-makers, government operational agencies, and funders, thus providing a holistic and integrative systems-thinking approach to address priority questions and achieve desired outcomes in One Health. The initial focus of OHA has been on infectious diseases, but there is increasing recognition of the potential benefits of the alliance to address broader complex issues. Greater involvement and overlap of the environmental sciences, human and animal health sciences, social science, and indigenous kaupapa Māori research is particularly critical for ensuring its success within the New Zealand context. Given the economic and cultural importance of New Zealand’s “*clean, green*” image, a One Health approach that draws strongly on the environmental sciences makes particular sense. Furthermore, as the global environment becomes increasingly stressed by anthropogenic pressures our research may hold potential solutions for similar challenges elsewhere.

## Introduction

There is growing recognition that complex health challenges at the human-animal-environmental interface require a transdisciplinary approach [1, 2]. In New Zealand, the response has taken the form of the One Health Aotearoa^1^ (OHA) alliance, established in response to a raft of increasingly complex anthropogenic pressures and growing health challenges that are difficult to address through a single discipline.

Despite several defining characteristics that make New Zealand an obvious place in which to undertake One Health research, our history has been to remain relatively siloed in our activities. This is particularly surprising given the indigenous Māori worldview and knowledge system that emphasises holism, transdisciplinarity and interconnectivity between humans, animals, and the environment.

In order to address this discrepancy, OHA was established with the explicit intention of breaking down traditional silos and bringing together leading New Zealand researchers from a wide range of disciplines and institutions, who can work collaboratively to build transdisciplinary capacity and innovation in partnership with stakeholders and policy-makers. OHA aims to be the national leader for research, education, and advocacy on health hazards at the human-animal-environmental interface, and to be the prime point of contact in New Zealand for engagement and collaboration in One Health. OHA is not simply a national One Health society, committee or interest group. The intention is to create a genuine culture change with the embedding of transdisciplinary thinking and action among researchers at a national level, running parallel with an indigenous worldview and with intrinsic and direct links to effective policy-making. We believe this initiative is novel globally because of these characteristics.

Here we describe the background, structure and aspirations of OHA

## One health: an obvious approach in the New Zealand context

The One Health approach is an obvious fit within the New Zealand context, and several characteristics suit a transdisciplinary, “whole of society” approach. These features include New Zealand’s relatively isolated ecosystem, strong economic dependency on agriculture and the physical environment, heavy reliance on fresh water, and a tight-knit scientific community well-aligned with an existing indigenous Māori worldview that emphasises holism and interconnectivity.

New Zealand’s ecosystem is defined by its physical island geography, a notable lack of indigenous terrestrial mammals, and comparatively late settlement by humans [3]. Quarantine measures were implemented early during the period of European colonisation to protect public health and the agricultural sector. Today, New Zealand’s biosecurity laws remain some of the strictest globally [3]. Consequently, New Zealand has relatively low rates of certain infections (e.g. bovine brucellosis has been eliminated and Q fever has never been reported), has the presence of strains rarely found elsewhere that arrived in the country many decades ago and have been evolving in isolation (e.g. *Campylobacter* [4] and Shiga toxin–producing *Escherichia coli* [5]), and experiences delayed impact of many infectious diseases from overseas [3]. One benefit of being a relatively “closed system” for some food and agricultural products is that the impact of health interventions can be evaluated in a more rigorous way that would not otherwise be possible. For example, campylobacteriosis rates were halved following an intervention that lowered contamination of fresh poultry [6–8]*.*

Conversely, New Zealand has a relatively high burden of certain infectious diseases. Leptospirosis remains an important occupationally-acquired infection in farmers and meat workers [9]. New Zealand also has an unusually high incidence of yersiniosis in humans [10], and salmonellosis [11], giardiasis [12], and cryptosporidiosis [12] are relatively common. The incidence of *Staphylococcus aureus* infections in New Zealand is among the highest reported in the developed world, with the highest incidence among Māori and Pacific Peoples [13]. The incidence of serious infectious diseases has also increased markedly in New Zealand over recent decades, and ethnic and social inequalities have also risen [14]. A major challenge for the country is to address the social, cultural, and environmental determinants of these high rates of infectious diseases and inequalities.

New Zealand also has high pet ownership, providing opportunities for transmission of certain zoonoses, with 64% of households owning companion animals [15]. Additionally, rates of international travel by New Zealand residents are among the highest globally, and net gain migration remains high [16]. Consequently, New Zealand remains vulnerable to pandemics and other global emerging disease threats.

Another defining characteristic of New Zealand is our strong economic reliance on agriculture, unusual among developed nations. New Zealand is experiencing some of the highest global rates of agricultural intensification [17], with implications for water quality and disease profile. While the global biomass ration of livestock to humans is ~ 2:1 [18], in New Zealand it is ~ 25:1 [19]. Agricultural intensification has been linked to water contamination [20], which has been associated with New Zealand’s high rates of zoonotic enteric disease [21]. Zoonoses associated with direct animal contact, such as *Salmonella* Brandenburg [22] and leptospirosis [23, 24], disproportionately affect farmers and meat workers, and people living in rural areas with large cattle populations are more likely to be infected with Shiga toxin-producing *E. coli* [25]. There is growing concern over the deteriorating quality of New Zealand’s natural environment, particularly fresh water quality [26]. Routes of transmission to humans via contaminated water include through irrigation of food crops, recreational activities, Māori customary resources, consumption of contaminated shellfish, as well as through drinking water. New Zealand’s disease profile at the human-animal-environmental interface is, therefore, likely to be different from many developed countries, characterised both by the need to deal with internal challenges and to resist external pressures from overseas. There is growing global focus on the health and environmental impacts of food production systems [27]. It is a challenge for a country that is so highly reliant on a narrow agricultural base to transition to a more sustainable and low risk agricultural system. Informing this transition may benefit from use of new, more comprehensive metrics that consider wider health, social, environmental, and economic impacts [28].

Finally, New Zealand’s scientific community is small and well-connected. New Zealand’s modest population of 4.9 million makes connecting with researchers, communities, and policy-makers relatively easy, while the centralised government system means fewer layers of bureaucracy to work through. Consequently, scientific research is relatively unified nationally, and standardised country-level interventions can be created, such as the New Zealand Antimicrobial Resistance Action Plan [29]. Examples of streamlined services include a national veterinary laboratory (The Animal Health Laboratory) and a centralised surveillance system for notifiable diseases (EpiSurv). The New Zealand Microbiology Network connects all clinical diagnostic microbiology laboratories across the country and facilitated nationwide initiatives with relative ease, such as a national surveillance study for Legionnaires’ disease [30]. New Zealand has a strong international reputation in biosecurity and food safety, and in 2016 established the New Zealand Food Safety Science and Research Centre as a nationwide partnership between government, the food industry and research organisations [31]. More broadly, the establishment of alliances across the health sector in parts of New Zealand has resulted in some of the most highly integrated health systems in the world [32].

Importantly, concepts of holism and interconnectivity between humans, animals and the environment are also reflected in an indigenous Māori worldview. Therefore, One Health is not necessarily a new epistemological concept in New Zealand; rather, it seeks to encourage new approaches and wider discussion to science and research that draws on previous perspectives, knowledges, and understandings, to promote new opportunities for sharing research and knowledge to understand increasingly complex systems and challenges that affect health. The One Health approach can, therefore, embrace societal and indigenous perspectives and values and, within this wider context, offers an opportunity to work closely with Māori to form a mutually beneficial partnership.

## One Health Aotearoa

OHA is an alliance of researchers whose central goal is to improve health and well-being in New Zealand by reducing the burden of infectious diseases and inequalities through integrated, cross-sectoral, and “whole-of-society” approaches to health hazards at the human-animal-environmental interface. A strong emphasis of OHA has been on facilitating the interactions of people from diverse disciplines and knowledges, linked to high-level engagement with policy-makers, government operational agencies, and funders. The intention is to use a more holistic and integrative systems-thinking approach to develop carefully targeted research questions and setting research priorities within an integrated framework. This provides a better platform for innovative, relevant and explicit research activities and opportunities. Importantly, issues are addressed in a real world context, with early involvement of key stakeholders to help co-design research, development of research questions, and easier translation of research findings into policy and actions.

Several national issues were catalysts for OHA, including a relatively high incidence of enteric infections, the potential adverse health effects of dairy intensification, threats to freshwater quality, and concerns about imported infectious diseases. In 2013, OHA was established with the aim of formalising existing connections, developing new research collaborations by offering a forum to discuss and align research priorities, and providing direct links to stakeholders, communities, and policy-makers. The initial focus of OHA has been infectious disease, but there is increasing awareness of the potential benefits of such a transdisciplinary alliance to address other issues, such as the effects of climate change and changing land use on ecosystem health. This has enabled a focus on health hazards at the human-animal-environmental interface, not just on zoonoses.

Although having no institutional boundaries, OHA was founded around a core alliance between New Zealand’s oldest medical school (University of Otago), New Zealand’s only veterinary school (Massey University), and the New Zealand Crown Research Institute of Environmental Science and Research (ESR), which is the main provider of infectious disease services to the Ministry of Health. OHA now engages researchers and professionals from many of New Zealand’s universities, crown research institutions, government agencies, and district health boards. There is also a heightened awareness that OHA should not be solely focused within New Zealand’s borders, and increasing efforts have been made to work with regional partners in the Pacific and Australia.

While OHA was originally established by the medical and veterinary professions, which have typically dominated One Health initiatives, there has always been ambition to widen traditional One Health philosophy and concepts to embrace other research disciplines and knowledges. We believe this approach to achieve transdisciplinarity is better placed to address a range of complex issues and to achieve desired sustainable development outcomes. This wider scope requires a strong cohesive and resourced alliance to facilitate change where interaction, collaboration, and integration become the norm across a broader range of disciplines and knowledges. OHA strives to aggregate the learnings from One Health, EcoHealth and Planetary Health domains and reflect their intent, mission and objectives, to facilitate inclusiveness and integrative approaches. Interconnection and interdependencies between the environment, human and animal health also lie at the heart of indigenous Māori epistemology. Understanding these intimate relationships and connections will require undertaking greater social science and kaupapa Māori^2^ research within One Health. A key learning from the 2013–2016 West African Ebola outbreak response was the early involvement of medical anthropologists who were instrumental in identifying key cultural and social practices that were contributing to transmission and impairing control efforts [33]. Likewise, integration of indigenous knowledge into environmental planning and decision making has been a core component of the management of water catchments in New Zealand [34, 35].

OHA further recognises the critical need to build meaningful relationships with indigenous Māori in One Health research in New Zealand and to identify their perspectives and priorities; to date, indigenous research has lacked depth and capacity. We also base this on the Treaty of Waitangi,^3^ which provides a foundation and principles in New Zealand on which to establish and build partnerships with Māori. The process adopted within OHA includes creating opportunities and advancing support for collaboration with Māori and Māori undertaking their own research.

Partnership with Māori is also a response to major health disparities in New Zealand, where Māori are over-represented in many health statistics, including infectious diseases [14]. OHA believes giving recognition to indigenous knowledge and values and respecting the importance of mātauranga Māori (knowledge created by Māori according to their experiences, history, worldview, values, culture and aspirations) is vital to its success. In terms of an integrative OHA philosophy, understanding the links between environment, human and animal health are significant for Māori. For example in the water quality area, mahinga kai (traditional Māori food and natural resources and the places they are sourced from) is a major customary activity impacted by agriculture and animals, which has great consequences for human health. Therefore, an OHA imperative is for greater partnership with Māori researchers and communities, to help co-design research to collectively address these types of issues. An ultimate aim is to improve health and wellbeing for Māori, and build capacity and diversity in the way we work, through using local knowledge next to science.

OHA has focused on forming a solid base around its founding institutions and raising its profile and influence throughout New Zealand, currently funded mainly from internal sources within founding institutions. It hosts a highly successful annual symposium held centrally in the nation’s capital city, bringing together a diverse range of researchers, professionals, government agencies, and policy-makers. OHA has successfully brought together a large and growing group of researchers and stakeholders who now know each other. It has grown and nurtured cross-discipline engagement to facilitate and guide new directions in collaborative research, increased dialogue between environmental, human health, and veterinary sciences, and engaged widely with government science advisors, professionals, international networks, and kaupapa Māori researchers. OHA is being increasingly recognised by government bodies and others as a national resource for expertise in infectious diseases and One Health, and OHA is now widely represented on key national committees and working groups. OHA has represented New Zealand on international initiatives such as the Oceania Planetary Health Forum, and aspires to be recognised as a centre of research excellence.

## Priority research areas

OHA has identified several research priorities, three of which have been developed into focussed work streams, or pou (meaning “pillars or central themes” in Māori language). The three central pou are: (1) antimicrobial resistance, (2) fresh water quality, and (3) emerging infectious diseases.

Interwoven through these three pou are three cross-cutting themes, to which all projects need to respond. The first is Vision Mātauranga, a Government science policy that aims to “unlock the innovation potential of Māori knowledge, resources and people for the benefit of Aotearoa-New Zealand.” [36] Vision Mātauranga is an important and integral cross-cutting component, guiding and contributing relevant research in all three central pou. The other cross-cutting themes are: (2) climate change and ecosystem disruption, and (3) achieving policy change, which includes One Health metrics, modelling and policy. These cross-cutting themes respond to the need to address globally important, human-made ecosystem threats within and across the main pou, and the need to carry out research on robust measures of impact and to identify ways for policy makers to make effective change.

Although New Zealand has low rates of antimicrobial resistance, the prevalence is growing [20, 37, 38] and the reasons for this increase, including sources and pathways of transmission, need to be understood [39–41]. Recent analyses show that antimicrobial use in animals is relatively low compared to other food trading countries in Europe, Australia, Canada, and USA [19], whereas human use is relatively high [42]. The New Zealand veterinary profession has set an aspirational goal to further reduce the use of antibiotics in animals, and phase out reliance on antimicrobials for the maintenance of animal health and welfare by 2030 [19].

Fresh water quality in New Zealand is declining, a trend closely tied to agricultural intensification [20, 38]. Greater understanding about points of contact and transmission of potential pathogens between animals, humans, and waterways is urgently needed. The importance of a One Health approach to this issue was demonstrated in 2016 with the massive outbreak of gastroenteritis in the town of Havelock North. The outbreak, one of the world’s largest reported waterborne outbreaks, was traced to sheep faeces contaminating bores supplying drinking water [43]. OHA researchers were at the forefront of the investigation and control of this outbreak.

The emergence and re-emergence of infectious diseases frequently require responses from multiple disciplines. Recent examples include leptospirosis [44], murine typhus [45], *Mycoplasma bovis* infection [46], *Salmonella enterica* Serovar Typhimurium DT160 infection [47], *E. coli* O157:H7 infection [48], and pandemic influenza [49]. These diseases have multiple pathways of entry. For example, *S*. Typhimurium DT160 was linked to wild birds, pandemic influenza was introduced by human travellers, and *M. bovis* was most likely brought in by material from cattle [46]. Concerns about the potential to introduce mosquito-borne and other diseases not currently established in New Zealand are real [50]. Understanding external drivers of imported infectious diseases is essential for informing biosecurity measures and pandemic preparedness [51]. New Zealand’s relative isolation may also provide opportunities to consider disease prevention options that are not available to larger, more connected geographical regions [52].

## Conclusion

New Zealand’s isolation, small population, unique natural environment, and growing aspiration for a healthy, well-managed and sustainable physical, economic, and social environment, makes it an excellent example of where a One Health approach makes sense, and where its scientific community can build a cohesive national-level alliance of researchers. Greater involvement and overlap of the environmental sciences, human and animal health sciences, social sciences, and indigenous kaupapa Māori-led research is critical for ensuring its success within the New Zealand context.

OHA has made great headway in breaking down traditional silos and better connecting with stakeholders and policy-makers. Despite an encouraging start, OHA still has a way to go to achieve its aspirational goals. The alliance must ensure it draws on a full range of relevant disciplines, knowledge systems, professional groups and community networks. The value of a One Health alliance is becoming increasingly recognised to researchers working within narrow subject areas as they grapple with a growing myriad of health, environmental and sustainability challenges. These challenges demand new ways of collaboration across boundaries and knowledges to define research priorities and find solutions that can achieve outcomes locally, nationally and internationally.

## Data Availability

Not applicable.

## Abbreviations

ESR: Institute of Environmental Science and Research
OHA: One Health Aotearoa

## Footnotes

1 Aotearoa is the indigenous Māori name for New Zealand.

2 A philosophical doctrine, incorporating the knowledge, skills, attitudes and values of Māori society.

3 The Treaty of Waitangi was signed in 1840 between iwi/hapū tribal Maori groups across New Zealand and the Crown and gives recognition to indigenous rights and equality. The Crown is the Queen of England and representatives being the New Zealand Government today.
